# P097: Antimicrobial usage in the treatment of patients with bloodstream infection

**DOI:** 10.1186/2047-2994-2-S1-P97

**Published:** 2013-06-20

**Authors:** AO Paula, ACD Oliveira, RF Rocha

**Affiliations:** 1 UFMG, Belo Horizonte, Brazil

## Introduction

Although indiscriminate use of broad-spectrum antibiotics is related to the occurrence of bacterial resistance, there is an abusive use of such drugs.

## Objectives

To determine the antimicrobial usage in the treatment of patients with bloodstream infection (BSI) caused by methicillin-resistant and susceptible *Staphylococcus aureus* (MRSA and MSSA, respectively).

## Methods

Retrospective cohort study performed in an Intensive Care Unit of a large hospital in Belo Horizonte. The population is comprised of patients diagnosed with *Staphylococcus aureus* BSI from 2007 to 2011. Data were obtained through patients’ medical records and Hospital Infection Control Committee. Therapy were categorized in: empirical treatment (before the culture test result) or directed (according to the BSI causing agent). Descriptive and univariate analysis were performed (using chi-square test or a Fisher's exact test). The hospital’s Ethics Committee approved the project.

## Results

62 patients were included, 31 in each group (MRSA and MSSA). The most common antibiotics prescribed for empirical treatment were vancomycin (69.4%), polymyxin B (46.8%), ertapenem (29.0%), meropenem (24.2%) and cefepime (3.2%). There was no significant difference between the groups analyzed and the class of antimicrobials empirically prescribed (p>0.05). For directed treatment, the antibiotics prescribed were vancomycin (45.2%) and methicillin (40.3%). On one hand, MRSA group used significantly more vancomycin (p=0,000). On the other hand, MSSA patients used more methicillin after the culture result (p=0,000).

## Conclusion

A large use of broad-spectrum antibiotics in empirical treatments was observed, given the hospital’s microbiological profile and the need to initiate appropriate treatment during the first 24 hours. However, the treatment targeting favored a rational use of antibiotics, reducing the action spectrum after culture results.

## Disclosure of interest

None declared

